# Kidney anatomy: three dimensional (3D) printed pelvicalyceal system models of the collector system improve the diagnosis and treatment of stone disease

**DOI:** 10.1590/S1677-5538.IBJU.2017.03.01

**Published:** 2017

**Authors:** Luciano A. Favorito

**Affiliations:** MD, PhD. Professor Associado da Unidade de Pesquisa Urogenital da Universidade do Estado de Rio de Janeiro.; Urologista do Hospital da Lagoa Federal, Rio de Janeiro

The May-June 2017 issue of the International Braz J Urol presents original contributions with a lot of interesting papers in different fields: Erectile Dysfunction, Renal Stones, Prostate Cancer, Renal Cell Carcinoma, Prostate Biopsy, Ureteroscopy, Hemorragic Cystitis, Retrograde Ejaculation, Intermittent Urethral Catheterization, Ureteropelvic Junction Obstruction, Laparoscopy, Vaginal Prolapse, BPH, Vesicoureteral and Renal Anomalies. Papers came from many different countries such as Brazil, USA, UK, Turkey, Korea, France, Taiwan, Greece, China, Italy, Germany, Israel and India, and as usual the editor’s comment highlights some papers. We decided to comment the paper about a very interesting topic: The use of pelvicalyceal system models in percutaneous nephrolithotripsy surgery.

Atalay et al. from Turkey reported on page 470 an interesting study about three dimensional (3D) printed pelvicalyceal system models on patient information before percutaneous nephrolithotripsy surgery. The authors studied patients with unilateral complex renal stones with indication of percutaneous nephrolithotripsy surgery. Usable data of patients were obtained from CT scans as Digital Imaging and Communications in Medicine (DICOM) format. Mimics software version 16.0 (Materialise, Belgium) was used for segmentation and extraction of pelvicalyceal systems. DICOM format were converted to stereolithography format. Finally, fused deposition modeling was used to create plasticine 3D models of pelvicalyceal systems. A questionnaire was designed for patients to assess personalized 3D models effect on patient’s understanding their conditions before percutaneous nephrolithotripsy surgery (PCNL). The day before surgery, each patient was seen by a urologist to deliver information about surgery. Questionnaire were answered by patients before and after presentation of 3D models and the results of the questions were compared. Results: Five patients’ anatomically accurate models of the human renal collecting system were successfully generated. After the 3D printed model presentation, patients demonstrated an improvement on their understanding of basic kidney anatomy by 60% (p=0.017), kidney stone position by 50% (p=0.02), the planned surgical procedure by 60% (p=0.017), and the complications related to the surgery by 64% (p=0.015). In addition, overall satisfaction of conservation improvement was 50% (p=0.02). They concluded that generating kidney models of PCSs using 3D printing technology is feasible, and understandings of the disease and the surgical procedure from patients were well appreciated with this novel technology.

The 3D printed technology is a new technology and had many applications in kidney surgery. Previous studies demonstrated the importance of 3D printing in the presurgical planning for laparoscopic and robotic partial nephrectomy (1, 2). In the present paper the authors showed a new application for this technique that will be very important in the relationship between the doctor and the patient.

Recently interesting studies demonstrated the importance of the kidney anatomy applied to training and execution of the flexible ureteroscopy (3, 4). Ureteroscopy surgical training programs use virtual reality simulators. In a recent paper we standardized the building of a three-dimensional silicone mold (cavity) of the collecting system, on the basis of polyester resin endocasts, which can be used in surgical training programs (5).

The two-part silicone mold is feasible, cheap and allows its use for exible ureteroscopy surgical training (5). The 3D printing technology is very precise to show the anatomy of the collector system. In the future this technology could be used to make 3D endocasts of the collector system and improve the surgical training programs of the flexible ureteroscopy and others endo urological kidney procedures.

Luciano A. Favorito, MD, PhD Professor Associado da Unidade de Pesquisa Urogenital da Universidade do Estado de Rio de Janeiro Urologista do Hospital da Lagoa Federal, Rio de Janeiro Editor Associado da International Braz J Urol

## References

[1] Wake N, Rude T, Kang SK, Stifelman MD, Borin JF, Sodickson DK (2017). 3D printed renal cancer models derived from MRI data: application in pre-surgical planning. Abdom Radiol.

[2] Rundstedt FC von, Scovell JM, Agrawal S, Zaneveld J, Link RE (2017). Utility of patient-specific silicone renal models for planning and rehearsal of complex tumour resections prior to robot-assisted laparoscopic partial nephrectomy. BJU Int.

[3] Marroig B, Fortes MA, Pereira-Sampaio M, Sampaio FJ, Favorito LA (2016). Two-part silicone mold. A new tool for flexible ureteroscopy surgical training. Int Braz J Urol.

[4] Marroig B, Frota R, Fortes MA, Sampaio FJ, Favorito LA (2016). Influence of the renal lower pole anatomy and mid-renal-zone classification in successful approach to the calices during flexible ureteroscopy. Surg Radiol Anat.

[5] Marroig B, Favorito LA, Fortes MA, Sampaio FJ (2015). Lower pole anatomy and mid-renal-zone classification applied to flexible ureteroscopy: experimental study using human three-dimensional endocasts. Surg Radiol Anat.

